# The relative age effect and the relationship between biological maturity and athletic performance in Austrian elite youth soccer players

**DOI:** 10.3389/fspor.2025.1583880

**Published:** 2025-04-17

**Authors:** Mirko Wenger, Robert Csapo

**Affiliations:** ^1^ Department of Sport and Human Movement Science, Centre for Sport Science and University Sports, University of Vienna, Vienna, Austria; ^2^ Sportklub Rapid, Vienna, Austria

**Keywords:** European football, youth athletes, biological age, talent identification, physical fitness, maturation

## Abstract

**Purpose:**

Publications from recent years suggest that the biological developmental stage of underage athletes has a significant impact on their athletic performance. This scientific study is the first quantitative attempt in Austria to investigate the impact of the relative age effect and biological maturity on the physical performance of young elite soccer players.

**Methods:**

Birth data from a total of 98 male players in the U13 to U18 age groups of a first-division Austrian soccer club were collected. By measuring height, sitting height and body mass, the individual biological age was calculated using the Mirwald equation. The athletes underwent a standardized battery of tests, which included assessments of speed, strength and endurance.

**Results:**

The results indicate a significant effect of the relative age effect on player selection in the U14 and U15 teams (*p* < .05), which diminishes with increasing player age. Compared to typical Austrian adolescents of similar age, U13, U14 and U15 players demonstrate a higher level of biological maturity (*p* < .05). Correlational analyses revealed that the maturity offset, reflecting the time before or after the greatest individual growth spurt, was positively related to eccentric hamstring strength (*r* = 0.82) and vertical jumping ability (*r* = 0.61) and positively related to sprint performance over 5, 10 and 20 m (0.62 < *r* < 0.69; all *p* ≤ .001).

**Conclusion:**

Biological maturity and the associated anthropometric adaptations are decisive for athletic performance. The study supports the notion that biologically more mature players achieve better athletic performance than less mature players, especially before the onset of puberty.

## Introduction

Modern academies of competitive youth soccer differ from health-related recreational sports in that they focus on identifying and developing underage talent (1). Supported by specially trained coaches and advanced training equipment, professional soccer clubs around the world aim to foster the development of young players to gain sporting and financial benefits (2, 3).

Thanks to the increasingly sport-specific training methods and individualized load management, the physical performance of professional soccer players has gradually improved in recent decades (4, 5). Similar developments can be observed in Austria, where youth competitive soccer players are now taller and heavier compared to their age-matched colleagues from the last decade and perform significantly better in performance-related tests for (reaction) speed, endurance, agility, coordination and (jumping) strength (6, 7).

The increased emphasis on physical development is also reflected in match demands. High-intensity actions, such as sprinting (>25.2 km/h) or high-speed running have become key determinants of sporting success in modern soccer (8). Consequently, physical performance is now prioritized alongside technical and tactical skills in player development.

At the same time, youth soccer players are typically grouped by calendar year, usually with a January 1st cut-off (9, 10). Although this system aims to promote fairness and equal opportunity, it can result in chronological age (CA) differences of up to twenty-four months within the same cohort.

Chronologically older children tend to be more advanced anthropometrically, physically and cognitively. Moreover, they may benefit from greater early sporting success, enhanced confidence and more playing time, which in turn leads to more practice opportunities, and increased motivation (11). These cumulative advantages may contribute to their overrepresentation in youth academies (3, 12). This pattern of selection bias based on birthdate is commonly referred to as the relative age effect (RAE), which describes the “asymmetry in birth date distribution that favors those players born early in the selection year and discriminates against participants born later in the year” (11). It has been robustly demonstrated across nations, age groups, and time spans, particularly in male youth competitive soccer (13–15).

However, recent studies suggest that biological maturity status, rather than CA or RAE alone, may better explain differences in anthropometry and athletic performance during puberty (16, 17). Unlike CA, which is determined by birthdate, biological age (BA) is inherently influenced by genetic and epigenetic factors (18, 19). This implies that not all adolescents commence or progress through the maturation process at the same time or rate (20, 21). For example, Johnson and colleagues (22) reported that skeletal age among youth soccer players of the same CA can vary by up to five years.

This biological variability has significant performance implications. More mature players tend to have advantages in height, body mass, strength or speed, and often outperform their less mature peers in key soccer tasks such as changes of direction, tackling or sprinting (23, 24). These maturity-related advantages increase their likelihood of being selected for youth academies (25).

To assess biological maturity, skeletal age estimation via X-ray of the left carpal bone is considered the gold standard. Alternatively, pubertal stage can be inferred from secondary sexual characteristics (26–28). However, both methods present practical and ethical limitations: X-ray involves a (low) dose of radiation, is costly and requires evaluation by experienced physicians, while assessments of secondary sexual characteristics raise significant privacy concerns and are rarely used in practice (29).

To address these challenges, Mirwald and colleagues developed the “maturity offset” (MO) method, a non-invasive and widely adopted technique for estimating biological maturity status from anthropometric data (30). This method, considered sufficiently valid and reliable for research purposes (29), calculates the time before or after peak height velocity (PHV) based on the date of birth, body mass, height and sitting height. In Austrian males, PHV typically occurs at 13.8 years (29). Hence, an MO of −0.5 years would correspond to a BA of 13.3 years, providing insight into an individual's current biological maturity status (30, 31).

Although the effects of relative age and biological maturity on youth soccer performance have been examined independently, few studies have considered their combined influence – particularly in the context of elite Austrian youth soccer. Moreover, little is known about how these factors relate to both physical performance and selection decisions within structured academy environments. By addressing both factors simultaneously in a real-world academy setting, this study aims to offer new insights into the interplay between maturity, performance, and selection in youth talent development. In line with recent findings (32–34), this study tests the hypothesis that chronologically older and biologically more mature players not only perform better in physical performance tests for strength, jumping ability, speed and endurance, but are also more likely to be selected into an Austrian elite soccer academy.

## Materials and methods

### Study design

This study used existing data from the 2022/2023 season, collected during routine assessments of youth teams at the academy of an Austrian first-division soccer club. Physiotherapists conducted the anthropometric measurements, while sports scientists were responsible for administering the performance tests. The testing was conducted by staff who were familiar with the standardized testing protocols and had prior experience administering these tests. Each member of the team involved in the testing held at least a bachelor's degree and had been affiliated with the club for a minimum of three years. All tests took place at the club's training facilities. While players ate at the academy twice a week, no additional dietary guidance was provided in relation to the individual tests.

### Participants

To be included in the study, participants had to be officially registered as players of the first-division club's (Bundesliga) soccer academy as of June 1st, 2022, and must have completed the most current standardized test battery. The birthday cut-off date for team assignment was January 1st, as dictated by regulations of the Austrian Soccer Federation and no athlete was represented on more than one team. The sample consisted of all eligible and available players from the mentioned youth teams, forming a convenience sample.

To capture a broad cross-section of the influence of biological maturity on performance, data were collected from five teams (U13, U14, U15, U16 and U18). In Austrian competitive soccer, the U18 team represents the oldest age group in the academy, hence no older teams were included. Teams younger than the U13 level were not considered, as our primary interest was in the interaction between RAE and biological maturity, which becomes more pronounced and performance-relevant as players approach and progress through puberty. All players were born between 2005 and 2010.

Eighteen U18 players were excluded due to injuries or because their commitments with youth national teams prevented their participation. Given the methodological challenges of assessing PHV in older players, a retest was not conducted (35).

Finally, the sample comprised a total of 98 participants distributed among the teams as follows: U13 (*n* = 23), U14 (*n* = 21), U15 (*n* = 23), U16 (*n* = 16), U18 (*n* = 15).

### Anthropometric measurements

At the start of the season in July and during the winter preparation period in January, the players' height, sitting height, and body mass were measured to the nearest of 0.5 cm and 0.1 kg by physiotherapists using calibrated scales (Soehnle, Style Sense Compact 300, Nassau, Germany), a portable stadiometer (Seca GmbH & Co. KG, Seca 217, Hamburg, Germany), and a standardized chair. The chair height was adjusted to ensure that, during the seat height measurements, participants could sit upright with their feet flat on the floor, thighs fully supported, and knees bent at a 90-degree angle. These measurements were used to calculate the body mass index, PHV and BA using the Mirwald et al. equation (30). CA at the time of the anthropometric testing, as well as the players' month and year of birth, were retrieved from the club's player database (Spectator Sports, SoccerLab, Hasselt, Belgium).

### Performance measurements and test protocol

Strength and jumping power tests were conducted separately for the U15, U16 and U18 teams in January, following a three-week winter break marking the start of the preparation period. Although all players had previously completed the test battery at least once in their careers, the procedures were explained in detail, and any questions were addressed by the sports scientists involved. Of note, for the purpose of this study, the jump and strength tests described above were also administered to U13 and U14 players, as these assessments are not part of the club's routine testing for younger age groups. These additional tests were conducted by the lead investigator at the end of the season, between late May and early June. All participants wore technical clothes provided by the club.

Players were scheduled alphabetically and attended the gym in 20-min intervals throughout the day between 9 am and 6 pm. To minimize potential variability caused by different shoe models, the jumping and strength tests were performed in socks (36). Players were allowed two attempts per test, with the best attempt recorded. If requested, a practice attempt was permitted. Following the guidelines of Markovic et al., a 60-s rest period was provided between trials (37).

After completing anthropometric measurements and a mobility assessment conducted by the team's physiotherapist, players performed a standardized 5-min warm-up on an ergometer (Matrix, CXP Target Training Cycle, Frechen, Germany) set at 100 watts. This was followed by a 5-minute individual preparation phase, during which most players performed preparation jumps, dynamic stretching, and an individual warm-up targeting the hamstrings.

Jumping tests were then performed on a force plate (VALD Performance, FDLite, Newstead, Queensland, Australia). Vertical jump height was assessed using the Counter-Movement Jump (CMJ), where players executed vertical jumps with their hands on their hips to minimize measurement errors (38). Each participant was instructed to start from an upright standing position, perform a rapid downward movement to a 90° knee angle, and simultaneously initiate the push-off.

In addition, the Reactive Strength Index (RSI) was measured through a hop test. The athletes were instructed to jump as high as possible while minimizing ground contact time. Players completed ten ankle jumps with minimal knee flexion while keeping their hands on their hips to minimize measurement errors. The best five jumps were averaged. The RSI was calculated based on jump height and ground contact time, with the result expressed in meters per second (m/s) (39).

Following the jump tests, strength measurements were conducted. Hamstring strength was assessed using a Nordic bench (VALD Performance, Nordboard, Newstead, Queensland, Australia) through the Nordic Hamstring Curl and Iso Prone tests. Each player completed one practice trial followed by two test attempts. During the Nordic Hamstring Curl, players were instructed to lower themselves from a kneeling position to the ground as slowly and evenly as possible over three seconds, maintaining a straight body, with their head up and chest lifted. Arms were crossed in front of the chest. The recorded values represent the average bilateral maximum force, measured in Newtons (N). In the Iso Prone test, players held a plank position with a 0° hip angle and elbows on the ground. Players were instructed to keep their legs straight and pull on the hooks while maximally tensing their hamstrings isometrically for three seconds. The bilateral isometric maximum force was similarly recorded in N.

Speed tests were conducted during the international break in April. To ensure optimal recovery, players were given three days off prior to the test and refrained from training or playing matches. Testing for all teams took place on the same Monday afternoon between 5 pm and 7 pm. Although all players had previously completed the test battery at least once in their careers, the procedure was thoroughly explained, and any questions were addressed by the sports scientists involved. A standardized 10-min warm-up, following the Raise, Activate, Mobilize, and Potentiate (RAMP) method, was led by the team's sports scientist (40). The athletes were instructed to perform each trial with maximal effort. To ensure optimal recovery, players were tested individually before proceeding to the next participant. A passive recovery period of 5 min was provided between the two attempts. Players were tested in alphabetical order. To minimize measurement errors through distinct reaction times or body position differences (41), players began the test in a standing position, 50 cm in front of the first timing gate (SmartSpeed Dash, VALD Performance, Newstead, Queensland, Australia). To reduce single-beamed timing errors caused by inappropriate height adjustments (42), the photocells were positioned at hip height (43). The athlete initiated the start independently upon receiving a signal from the sports scientist. Wearing soccer shoes, they sprinted on artificial turf. Split times were recorded at 5, 10, and 20 m, with each player completing two attempts. The fastest time for each section was used for analysis.

Aerobic capacity was assessed for all teams during the final week of June, on a Monday afternoon between 5pm and 7pm just before the season's conclusion, using the Intermittent Fitness Test proposed by Buchheit and Rabbini (44). To ensure optimal recovery, players were given two days off prior to the test and refrained from training or playing matches. Following the recommendations of the developers, players performed a light individual warm-up and stretching routine before starting the endurance test (44). The procedure was explained in detail by the responsible sports scientist, and any questions were addressed. All players from each team completed the test simultaneously and the players were instructed to complete as many stages as possible. Conducted on artificial turf with players wearing soccer shoes, the test continued until the player could no longer maintain the required pace or failed to reach the 3-m zone three consecutive times. The final speed at which the player stopped was recorded and divided by a factor of 1.2 to calculate the maximum aerobic speed (MAS), expressed in km/h.

### Academy training structure and long-term player development

Players typically join the academy at the earliest opportunity, with many having been part of the club since the U9 or U10 age groups. By this stage, most players already have experience playing for lower-level clubs. Training for U9, U10 and U11 players takes place three times a week for a total of 4.5 h and emphasizes a multi-sport approach, including different ball games, track and field activities, and gymnastics to promote general motor skill development and coordination. In the U12 age group, the training frequency increases to four sessions for a total of 5 h per week. By the time players enter the U13 team, those who joined the club at the U9 level have accumulated four years of multi-sport training experience.

From the U13 level onwards, training shifts toward more specific soccer-related training. U13 and U14 players train four times per week for a total of 6 hours, including one gym-based session dedicated to age-appropriate strength training. At the U15 and U16 levels, training frequency increases to six sessions per week for a total of 9 h, with two strength sessions designed to develop basic strength and technique, and increase muscle cross-sectional area.

For U18 players, training intensity and specificity peak, with seven weekly sessions for a total of 10 hours, including two strength sessions targeting hypertrophy and maximum strength development, depending on the season's phase.

Up to the U14 level, teams participate exclusively in weekly friendly matches or tournaments organized by the club rather than a dedicated league. From the U15 level onwards, players compete in a nationwide league organized by the Austrian Football Association, with each club playing a total of 22 matches. The season runs from late August to May, with matches typically held on Saturdays. Three-week breaks occur in December and June, while January, February, July, and August are designated as preparation periods.

### Data processing and statistical analyses

To examine the RAE in all teams, Chi^2^ tests were employed to assess the distribution of players across birth quartiles. If the results indicated significant deviations from a uniform distribution, Cramer's *V* was computed to quantify the effect size. Comparisons of the respective test statistics between teams provided insight into potential variations in the RAE across different age groups.

According to a study by Müller and colleagues the age of PHV determined by carpal bone X-ray in Austrian adolescents is 13.8 ± 0.4 years (29). Using this information, an expected MO for each team was calculated as the difference between the average player age expected in the absence of RAE and the average age of PHV of male Austrians. Thus, the expected MOs were −1.3 (12.5–13.8), −0.3 (13.5–13.8), 0.7 (14.5–13.8), 1.7 (15.5–13.8) and 3.2 (17–13.8) years in the U13, U14, U15, U16 and U18 teams, respectively. While classical RAE studies often compare the distribution of players' birthdates to that of the general population, such population-level reference data (by birth month or quarter and year) were not available for this study. Therefore, RAE effects were operationalized indirectly by comparing the players' actual MO and BA to team-level expected values under the assumption of a uniform age distribution. To assess whether player selection was influenced by both RAE and the pace of biological maturation, the players' actual MOs were calculated using the Mirwald equation and compared to these theoretical values using one-sample *t*-tests (30). When significant results were found, Cohen's *d* was reported as measure of effect size. Raincloud boxplots, grouped by team, were generated to visually illustrate potential differences between expected and actual MOs.

To further isolate the effects of the pace of biological maturation, the difference between the players' actual BA – derived from the difference between CA and actual MO – and the expected BA (12.5, 13.5, 14.5, 15.5, and 17 years, respectively) was computed. These differences were then evaluated against the expected value of 0 years using additional one-sample *t*-tests, with Cohen's *d* calculated for effect size in cases of significant results.

To examine potential differences in performance metrics between players born in different birth quartiles, one-way ANOVAs were conducted for each outcome variable using pooled data from all age groups.

Finally, Pearson correlation coefficients and scatter plots were used to examine the relationship between the MO, performance measures and anthropometric data collected. All statistical analyses were performed using R (version 4.3.0, R Core Team, 2023). A significance level of *α* < .05 was set for all tests.

## Results

Descriptive statistics reflecting the players' CA and estimated BA as well as anthropometric characteristics are shown in Table 1.

**Table 1:** Players’ CA and estimated BA as well as anthropometric characteristics.

Team	*n*	CA (yrs)	BA (yrs)	Height (cm)	Mass (kg)	BMI (kg·m^−2^)
U13	23	12.9 ± 0.3	13.2 ± 0.7	162.1 ± 9.2	47.1 ± 7.7	17.8 ± 1.6
U14	21	13.9 ± 0.3	14.1 ± 0.7	166.5 ± 9.2	54.7 ± 10.5	19.5 ± 1.9
U15	23	14.6 ± 0.3	15.1 ± 0.5	176.1 ± 7.1	63.8 ± 6.8	20.6 ± 1.7
U16	16	15.0 ± 0.3	15.2 ± 0.9	172.2 ± 7.9	63.3 ± 7.8	21.3 ± 1.6
U18	15	16.5 ± 0.6	16.6 ± 0.6	176.1 ± 7.1	67.6 ± 7.7	21.8 ± 2.3

CA, chronological age; BA, biological age; BMI, body mass index.

**Figure 1:**
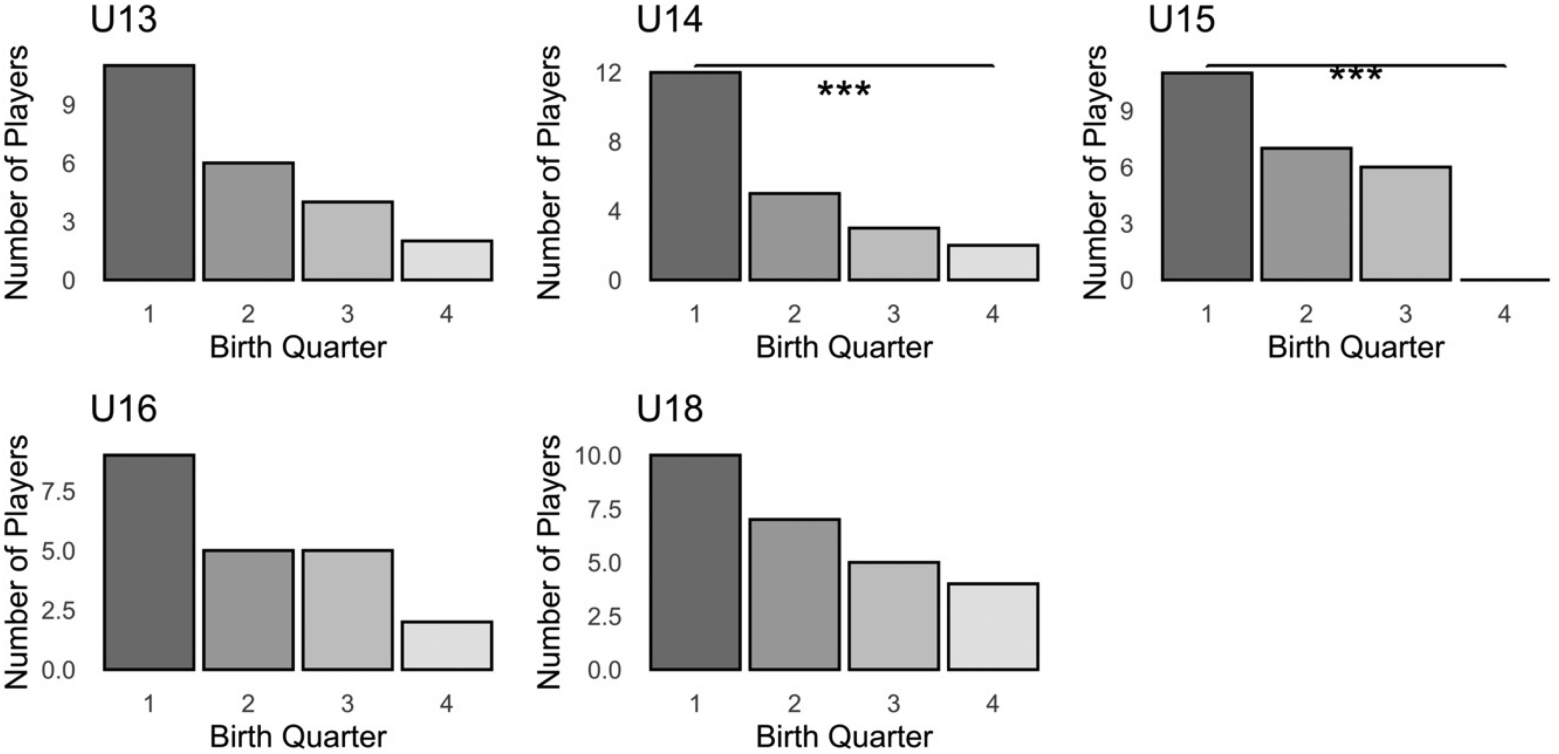
Distribution of players born in different birth quarters across teams. *** indicate significant deviations from a uniform distribution.

Across the entire sample, the distribution of players born in different birth quarters was notably left-skewed (*χ*(3) = 33.586, *p* ≤ .001, *V* = .311). The team-specific analyses are depicted in Figure 1. While the U13 team results did not reach significance, statistically significant deviations from a uniform distribution were observed in the U14 and U15 teams (*χ*^2^_U13_(3) = 7.783, *p* = .051; *χ*^2^_U14_(3) = 11.091, *p* = .011, *V* = .410; *χ*^2^_U15_(3) = 10.333; *p* = .016, *V* = .379). In the older teams, the RAE was less pronounced, resulting in non-significant findings (*χ*^2^_U16_(3) = 4.714, *p* = .194; *χ*^2^_U18_(3) = 3.231; *p* = .357).

**Figure 2:**
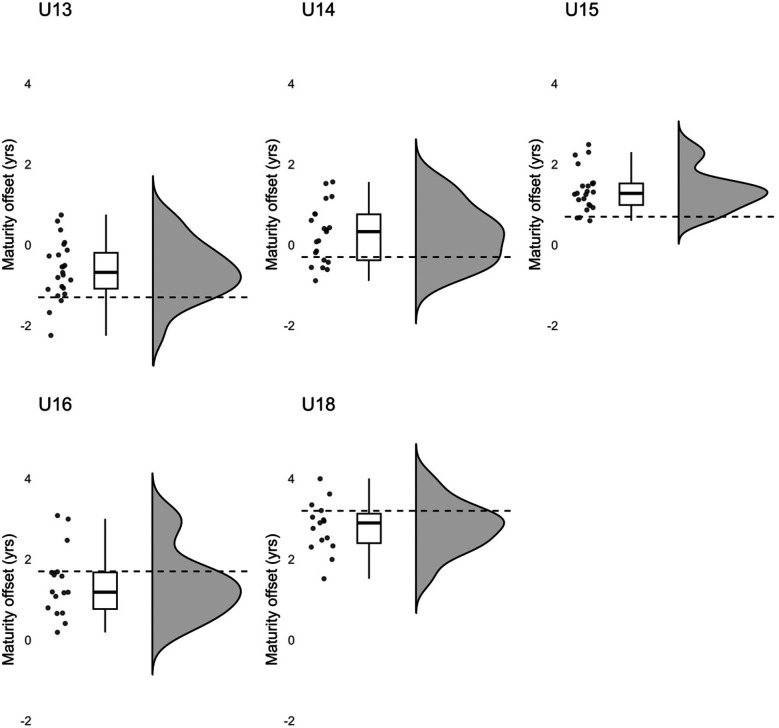
Expected (dashed line) and observed maturity offsets across teams.

Comparisons of MOs revealed substantial differences between expected (U13 = −1.3; U14 = −0.3; U15 = 0.7; U16 = 1.7 and U18 = 3.2) and observed values (see Figure 2). The players in the U13, U14, U15 and U16 teams were consistently biologically older than expected, with one-sample *t*-tests confirming significant differences in the U13, U14 and U15 teams (*t*_U13_(22) = 4.399, *p* < .001, *d* = .917; *t*_U14_(20) = 3.572, *p* = .002, *d* = .780; *t*_U15_(22) = 6.043, *p* < .001, *d* = 1.260; *t*_U16_(15) = 1.393, *p* = .184). In the U18 age group, however, the actual MOs were significantly smaller than the expected values (*t*_U18_(14) = −2.457, *p* = .028, *d* = −.634).

Comparing the differences between CA and BA against an expected value of 0 years, one-sample *t*-tests revealed significant disparities within the total sample, which contained data pooled from all teams (*M* = −0.27, *SD* = ±0.64; *t*_total_(97) = −4.162, *p* < .001, *d* = −.420), as well as in the U15 category (*M* = −0.59, *SD* = ±0.46; *t*_U15_(22) = −6.123, *p* < .001, *d* = −1.277). Although statistical significance was not reached in the other teams, descriptive statistics indicate that players exhibited a biological development beyond what their CA predicted (*M*_U13_ = −0.24, *SD*_U13_ = ±0.65; *M*_U14_ = −0.19, *SD*_U14_ = ±0.69; *M*_U16_ = −0.16, *SD*_U16_ = ±0.75; *M*_U18_ = −0.07, *SD*_U18_ = ±0.60).

One-way ANOVAs, conducted on the pooled data from all teams, to assess differences in body height, body mass, and performance metrics across birth quarters revealed no statistically significant results (0.378 < *p* < 0.935).

**Figure 3:**
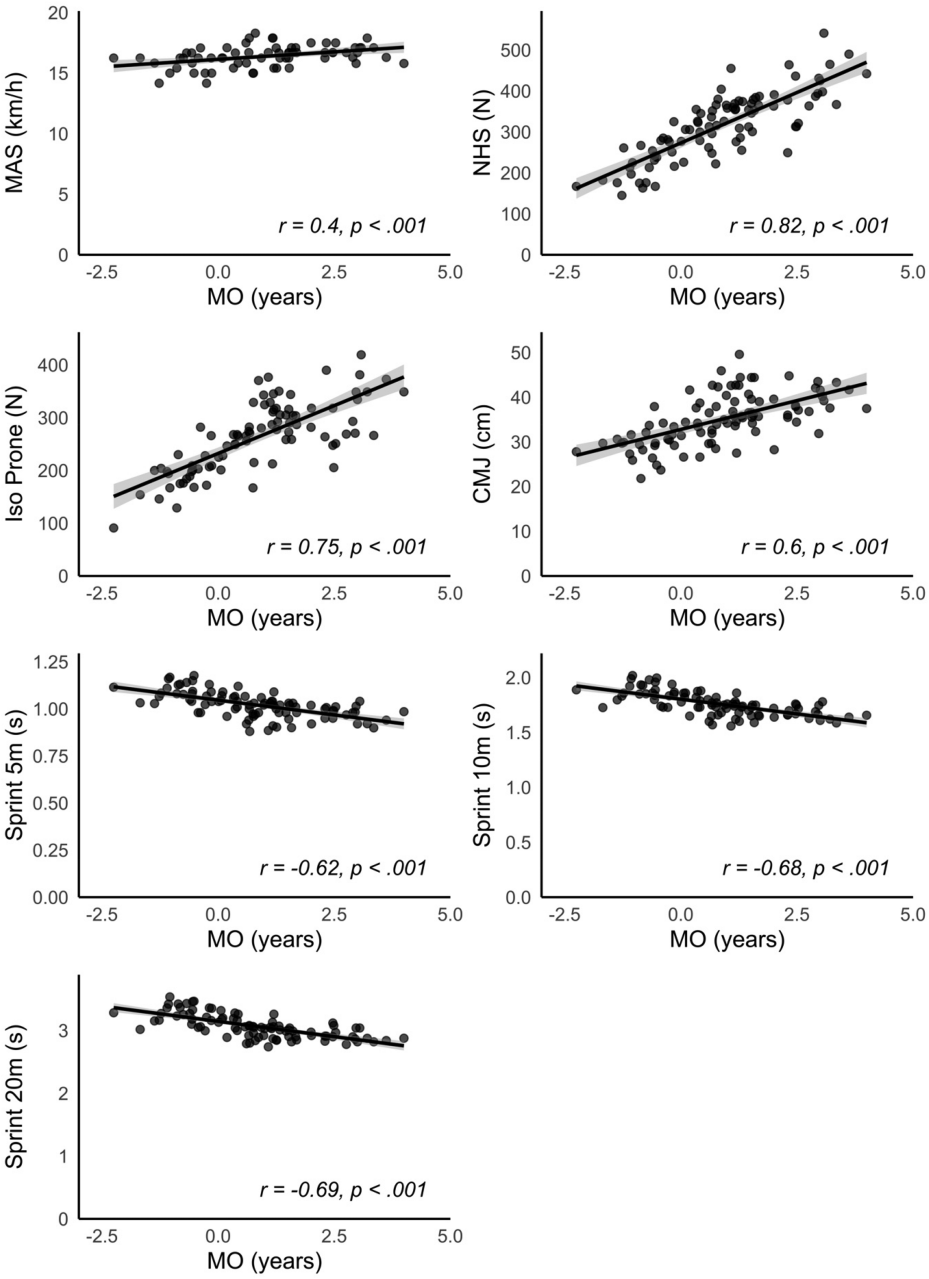
Relationship between maturity offset (MO) and performance test results conducted across the entire group. MAS, maximal aerobic speed; NHS, nordic hamstring strength; CMJ, countermovement jump height, 5 m/10 m/20 m: sprint times over 5, 10 and 20 m, respectively.

**Figure 4:**
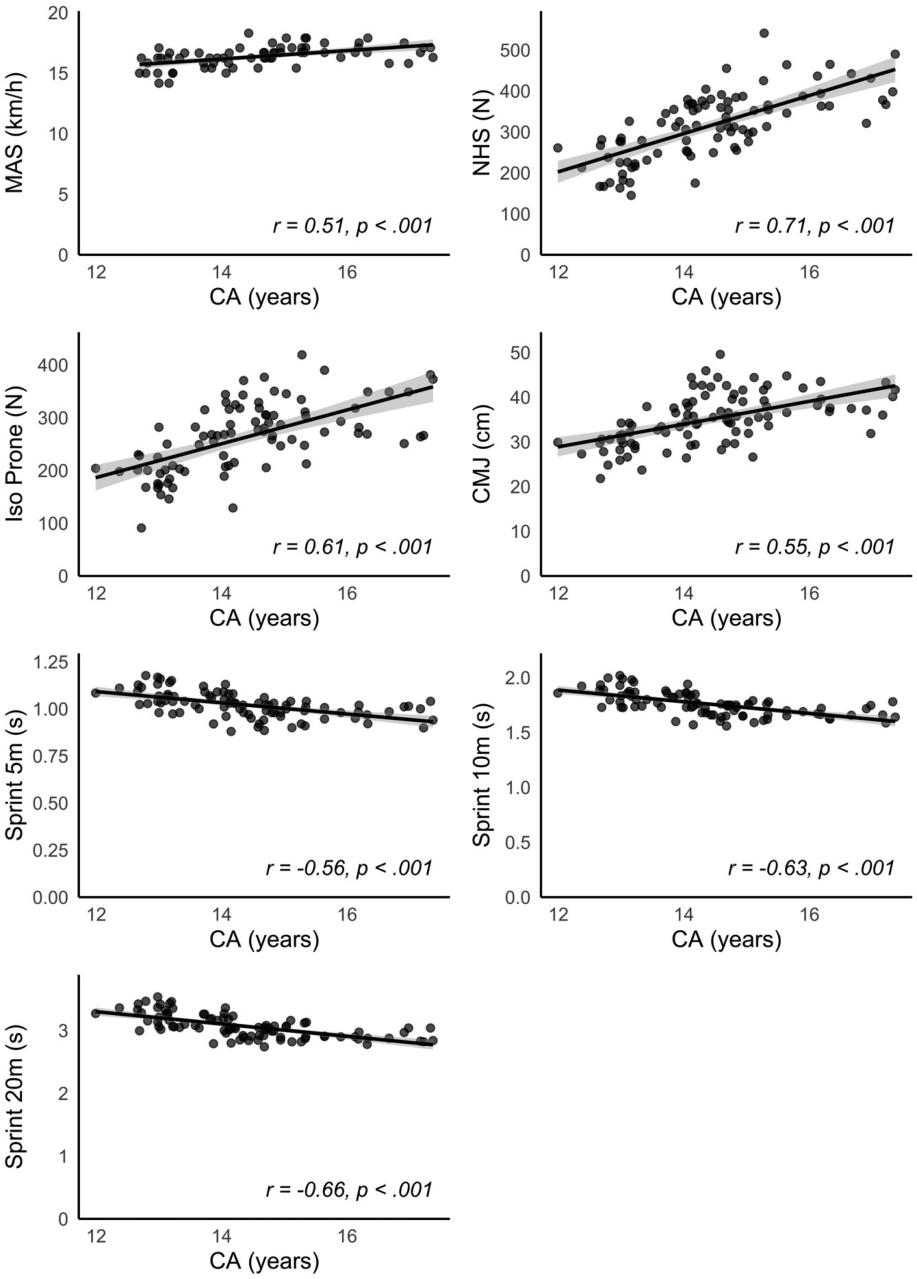
Relationship between chronological age (CA) and performance test results conducted across the entire group. MAS, maximal aerobic speed; NHS, nordic hamstring strength; CMJ, countermovement jump height, 5 m/10 m/20 m: sprint times over 5, 10 and 20 m, respectively.

The correlational analyses unveiled strong associations between MO and eccentric and isometric hamstring strength, as well as CMJ height, while exhibiting a positive correlation with sprint performance over 5, 10 and 20 m (*r* > .50; *p* < .001). An average positive correlation was found between MO and RSI as well MAS (.30 < *r* < .50; *p* < .001). The corresponding scatter plots illustrating the relationships between MO and performance parameters are shown in Figure 3. For comparison, Figure 4 presents the same correlations using CA instead of MO. With the exception of MAS, for which the correlation was slightly higher with CA, all other performance variables showed consistently stronger correlations with MO than with CA.

## Discussion

This study aimed to evaluate the influence of the RAE on player selection within the youth teams of an elite Austrian soccer club. We hypothesized that chronologically older and biologically more mature players would not only perform better in physical performance tests for strength, jumping ability, speed and endurance, but also be more likely to be selected into an elite soccer academy. Our findings support this hypothesis. A clear RAE was observed, particularly in the younger age groups, and biological maturity showed consistent associations with key physical performance parameters. These results highlight the combined impact of relative age and biological maturity on both selection and performance within the structured academy setting.

While previous studies have investigated the presence of the RAE in elite youth soccer, this study provides novel insights within the context of Austrian elite soccer. For the first time, we offer data on the current state of youth elite soccer in Austria, incorporating a comprehensive range of physical performance tests within the same population. These assessments cover aerobic endurance, isometric and eccentric strength, explosive power, and neuromuscular capacity through jumping and sprinting measurements. Furthermore, the study examines five distinct age groups from the same club environment, allowing for a unique analysis of how biological maturity correlates with performance metrics and how these relationships evolve across different developmental stages.

Our findings clearly demonstrate the presence of the RAE, as, across teams, there was a significant overrepresentation of players born in the first quarter of their respective selection periods, starkly contrasting with the uniform distribution of births per month in the general Austrian population. The analysis of the individual teams showed that within the U13, U14 and U15 teams there was a birth ratio of 3:1 favoring of players born in the first semester, although this ratio decreased to approximately 2:1 in the U16 and U18 age groups. Notably, in the U15 team there was not a single player born in the last quarter of the year. Our results are consistent with those reported in studies conducted in other European countries, which have documented similar distributions in youth soccer academies over the last 15 years (10, 11, 45).

The diminishing impact of the RAE observed from U16 level onwards suggests that scouting and the player selection are especially influenced by the RAE in pre- and peripubertal boys (33, 46). This phenomenon could be attributed to the advantage of older players, who may compensate for technical, tactical or psychological shortcomings with superior physical attributes before puberty. However, as their teammates catch up during the growth spurt phase, these players may ultimately be identified and excluded due to potential skill deficits. Conversely, it is plausible that younger players, who may initially lag behind in performance, experience improvements after puberty. Consequently, they might have to leave the academy less often after reaching this stage.

Even though the players studied reached their age of PHV at 13.7 years, which aligns with both that reported in other studies in youth soccer players and that found in general Austrian adolescents (29, 47, 48), our results indicate that especially in younger age, the academy tends to preferentially accept early biological developers. Indeed, across all teams, the players’ average BA consistently exceeded their CA, although in the team-specific analyses significant differences were only observed in the U15 team.

The findings indicate that biological maturity status, rather than birth quarter, plays a more significant role in individual sports performance, as players with advanced maturity tend to outperform their less mature peers. With respect to the connections between biological maturity and performance metrics, the correlational analyses and scatter plots revealed significant linear relationships between MO and strength and speed parameters, such as eccentric and isometric strength of the hamstrings, vertical jumping ability and speed over 5, 10 and 20 m. Previous research suggests that more than a quarter of the variance in sprint performance among competitive youth soccer players can be explained by eccentric strength as measured by relative Nordic hamstring power (49).

Our findings underscore the advantages conferred by advanced biological maturity and the associated anthropometric adaptations in improving strength and speed parameters, which are crucial for physical contact sports like youth soccer. These attributes are strongly correlated to success metrics and playing time (50–52). Interestingly, the Austrian soccer academy system operates as a closed system, with no relegation among its twelve members. While the system aims to foster the development of all players, our data suggest that current selection patterns may unintentionally favor chronologically older or biologically more mature players. This could reflect a tendency to prioritize short-term performance over long-term development. However, without comparison to unselected players, it is not possible to fully distinguish between selection bias and sport-inherent demands that naturally favor more physically developed athletes.

To mitigate the developmental disadvantages faced by relatively younger or late-maturing players within such systems, several alternative strategies have been proposed. One practical approach to addressing these challenges is bio-banding, which groups athletes based on their biological rather than chronological age (53, 54). Bio-banding aims to create more equitable and developmentally appropriate opportunities for all players, regardless of their birth date or maturation rate (55, 56). At the club level, bio-banding can group U13, U14 and U15 players by biological rather than chronological age for soccer and strength training. Organizing friendly matches or tournaments between bio-banded teams, alongside official competitions, ensures that less biologically developed players receive ample playing time and skill development opportunities. Scouts, coaches, and relevant personnel should regularly reassess their selection criteria and receive ongoing education on the RAE, BA, and their impact on short- and long-term player development (57).

In conclusion, the presence of the RAE was observed among young players in the academy of an elite Austrian soccer club. During the prepubertal age, differences in both CA and the pace of biological development can result in differences of several years in biological maturity. Our findings underscore the interconnectedness of advanced biological maturity and key sports motor skills such as speed and strength, which are crucial for youth soccer performance. Since sporting talent is not tied to one's birth month, it is evident that the current setup systematically excludes numerous talented but later-born or less-developed players from the system at a young age (58).

## Limitations

While the performance tests were carried out according to a standardized test protocol, it cannot be ensured that all tests were conducted under identical conditions, including timing, explanation, and execution of movements, owing to variations among test instructors. Discrepancies in the accuracy of the measurement of some parameters may have occurred, potentially leading to unprecise calculations of the age of PHV and, consequently, biological maturity. The fact that the players' nutritional status was not assessed may have introduced variability in the testing outcomes. The study is also limited due to the small sample sizes in each group, which is why the reported effect sizes should be interpreted with caution. Several authors have noted that the Mirwald equation tends to overestimate the age of PHV in early-maturing children, while underestimating it in late developers (59, 60). This systematic bias can result in a regression toward the mean, potentially misclassifying individuals at the extremes of the maturity spectrum. The method also appears to be more accurate in younger adolescents (approximately 10–13 years), which may reduce its accuracy when applied to older age groups who are closer to or beyond their age of PHV. In addition to these statistical limitations, it is important to recognize that most anthropometry-based equations – including those by Mirwald, Khamis-Roche, and Fransen – were developed in largely homogeneous, predominantly Caucasian populations. This raises questions about the validity of applying these methods to the ethnically diverse groups typically found in elite youth soccer settings. As Fransen (2021) recommended, such estimations should be interpreted with caution when used outside their original reference populations (61). Other non-invasive, anthropometry-based methods face similar limitations in accuracy and produce similar estimates of BA (62), although recent equations such as the one proposed by Fransen and colleagues (63) may help mitigate some of the known biases of earlier models. More precise techniques, such as skeletal age assessment via X-ray or the evaluation of secondary sexual characteristics, were not feasible in our setting due to ethical, logistical, and privacy-related constraints, and promising newer alternatives such as skeletal ultrasound were not available. Finally, it is important to note that the RAE may vary between countries. Therefore, caution is warranted when extrapolating the findings of this study, which focuses exclusively on Austrian athletes, to populations in other countries.

## Data Availability

The datasets presented in this study can be found in online repositories. The names of the repository/repositories and accession number(s) can be found in the article/Supplementary Material.
